# 4-Chloro-2-methyl-*N*-(4-methyl­phen­yl)benzene­sulfonamide

**DOI:** 10.1107/S1600536810032320

**Published:** 2010-08-18

**Authors:** B. Thimme Gowda, Sabine Foro, P. G. Nirmala, Hartmut Fuess

**Affiliations:** a Department of Chemistry, Mangalore University, Mangalagangotri 574 199, Mangalore, India; b Institute of Materials Science, Darmstadt University of Technology, Petersenstrasse 23, D-64287 Darmstadt, Germany

## Abstract

The asymmetric unit of the title compound, C_14_H_14_ClNO_2_S, contains two independent mol­ecules. The torsion angles of the C—SO_2_—NH—C segments in the two mol­ecules are −76.5 (5) and −48.3 (4)°. The two aromatic rings are tilted relative to each other by 76.6 (2)° in one mol­ecule and 70.7 (2)° in the other. In the crystal structure, inter­molecular N—H⋯O hydrogen bonds link the mol­ecules into centrosymmetric dimers.

## Related literature

For the preparation of the title compound, see: Savitha & Gowda (2006 ▶). For our studies of the effect of substituents on the structures of *N*-(ar­yl)aryl­sulfonamides, see: Gowda *et al.* (2009 ▶, 2010 ▶). For related structures, see: Gelbrich *et al.* (2007 ▶); Perlovich *et al.* (2006 ▶).
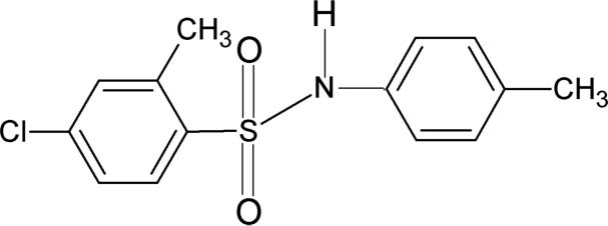


## Experimental

### 

#### Crystal data

C_14_H_14_ClNO_2_S
*M* *_r_* = 295.77
Monoclinic, 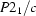

*a* = 10.544 (1) Å
*b* = 10.674 (1) Å
*c* = 25.196 (3) Å
β = 96.83 (1)°
*V* = 2815.6 (5) Å^3^
*Z* = 8
Cu *K*α radiation
μ = 3.77 mm^−1^
*T* = 299 K
0.30 × 0.30 × 0.25 mm

#### Data collection

Enraf–Nonius CAD-4 diffractometer
Absorption correction: ψ scan (North *et al.*, 1968 ▶) *T*_min_ = 0.398, *T*_max_ = 0.453
5050 measured reflections
4826 independent reflections
3917 reflections with *I* > 2σ(*I*)
*R*_int_ = 0.086
3 standard reflections every 120 min  intensity decay: 2.4%

#### Refinement

*R*[*F* ^2^ > 2σ(*F* ^2^)] = 0.097
*wR*(*F* ^2^) = 0.317
*S* = 1.43
4826 reflections
347 parameters
H-atom parameters constrained
Δρ_max_ = 1.01 e Å^−3^
Δρ_min_ = −1.01 e Å^−3^

### 

Data collection: *CAD-4-PC* (Enraf–Nonius, 1996 ▶); cell refinement: *CAD-4-PC*; data reduction: *REDU4* (Stoe & Cie, 1987 ▶); program(s) used to solve structure: *SHELXS97* (Sheldrick, 2008 ▶); program(s) used to refine structure: *SHELXL97* (Sheldrick, 2008 ▶); molecular graphics: *PLATON* (Spek, 2009 ▶); software used to prepare material for publication: *SHELXL97*.

## Supplementary Material

Crystal structure: contains datablocks I, global. DOI: 10.1107/S1600536810032320/bt5325sup1.cif

Structure factors: contains datablocks I. DOI: 10.1107/S1600536810032320/bt5325Isup2.hkl

Additional supplementary materials:  crystallographic information; 3D view; checkCIF report

## Figures and Tables

**Table 1:** Hydrogen-bond geometry (Å, °)

*D*—H⋯*A*	*D*—H	H⋯*A*	*D*⋯*A*	*D*—H⋯*A*
N1—H1*A*⋯O4^i^	0.86	2.02	2.874 (5)	171
N2—H2*A*⋯O2^i^	0.86	2.23	2.968 (5)	144

Symmetry code: (i) 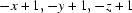.

**Fig. 1.**
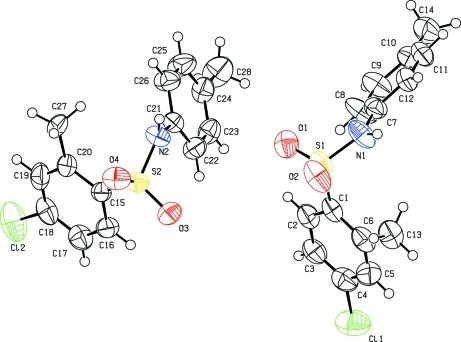
The molecular structure of the title compound, showing the atom labelling scheme and displacement ellipsoids are drawn at the 50% probability level.

**Fig. 2.**
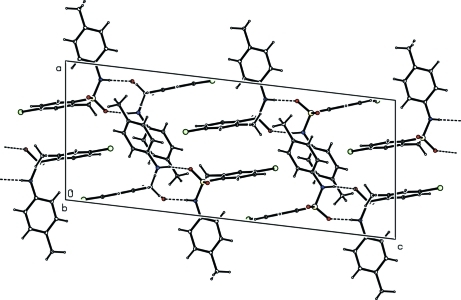
The molecular packing of the title compound with hydrogen bonding shown as dashed lines.

## supplementary crystallographic information

### Comment

As part of a study of the substituent effects on the crystal structures of *N*-(aryl)arylsulfonamides (Gowda *et al.*, 2009, 2010), in the present work, the structure of 4-chloro-2-methyl-*N*-(4-methylphenyl)benzenesulfonamide (I) has been determined (Fig. 1). The asymmetric unit of (I) contains two independent molecules. In one of the molecules, the conformation of the N—C bond in the C—SO_2_—NH—C segment has *gauche* torsions with respect to the S═O bonds. The conformation of the N—H bonds are *syn* to the *ortho*-methyl groups in the sulfonyl benzene rings of both the molecules, similar to that observed in 4-chloro-2-methyl-*N*-(phenyl)benzenesulfonamide (II) (Gowda *et al.*, 2009) and 4-chloro-2-methyl-*N*-(4-chlorophenyl)benzenesulfonamide (III) (Gowda *et al.*, 2010).

The torsion angles of the C—SO_2_—NH—C segments in the two molecules of (I) are -76.5 (5)° and -48.3 (4)°, compared to the values of -61.9 (4)° and 69.7 (4)° in the two independent molecules of (II) and 55.0 (2)° in (III).

The sulfonyl and the aniline benzene rings in (I) are tilted relative to each other by 76.6 (2)° in molecule 1 and 70.7 (2)° in molecule 2, compared to the values of 86.6 (2)° and 83.0 (2)° in the two independent molecules of (II) and 67.0 (1)° in (III).

The other bond parameters in (I) are similar to those observed in (II), (III) and other aryl sulfonamides (Perlovich *et al.*, 2006; Gelbrich *et al.*, 2007).

In the crystal, the intermolecular N—H···O hydrogen bonds (Table 1) link the molecules into infinite zigzag row like chains. Part of the crystal structure is shown in Fig. 2.

### Experimental

The solution of *m*-chlorotoluene (10 ml) in chloroform (40 ml) was treated dropwise with chlorosulfonic acid (25 ml) at 0° C. After the initial evolution of hydrogen chloride subsided, the reaction mixture was brought to room temperature and poured into crushed ice in a beaker. The chloroform layer was separated, washed with cold water and allowed to evaporate slowly. The residual 2-methyl-4-chlorobenzenesulfonylchloride was treated with 4-methylaniline in the stoichiometric ratio and boiled for ten minutes. The reaction mixture was then cooled to room temperature and added to ice cold water (100 cc). The resultant solid 4-chloro-2-methyl-*N*-(4-methylphenyl)benzenesulfonamide was filtered under suction and washed thoroughly with cold water. It was then recrystallized to constant melting point from dilute ethanol. The purity of the compound was checked and characterized by recording its infrared and NMR spectra (Savitha & Gowda, 2006).

The prism like colourless single crystals used in X-ray diffraction studies were grown in ethanolic solution by slow evaporation at room temperature.

### Refinement

The H atoms were positioned with idealized geometry using a riding model [C—H = 0.93–0.96 Å] and were refined with isotropic displacement parameters (set to 1.2 times of the *U*_eq_ of the parent atom).

The residual electron-density features are located in the region of O1 and S2. The highest peak is 0.77 Å from O1 and the deepest hole is 0.83 Å from S2.

### Crystal data

**Table:**

C_14_H_14_ClNO_2_S	*F*(000) = 1232
*M**_r_* = 295.77	*D*_x_ = 1.395 Mg m^−^^3^
Monoclinic, *P*2_1_/*c*	Cu *K*α radiation, λ = 1.54180 Å
Hall symbol: -P 2ybc	Cell parameters from 25 reflections
*a* = 10.544 (1) Å	θ = 5.9–19.1°
*b* = 10.674 (1) Å	µ = 3.77 mm^−^^1^
*c* = 25.196 (3) Å	*T* = 299 K
β = 96.83 (1)°	Prism, colourless
*V* = 2815.6 (5) Å^3^	0.30 × 0.30 × 0.25 mm
*Z* = 8	

### Data collection

**Table:**

Enraf–Nonius CAD-4 diffractometer	3917 reflections with *I* > 2σ(*I*)
Radiation source: fine-focus sealed tube	*R*_int_ = 0.086
graphite	θ_max_ = 66.9°, θ_min_ = 3.5°
ω/2θ scans	*h* = −12→12
Absorption correction: ψ scan (North *et al.*, 1968)	*k* = −12→1
*T*_min_ = 0.398, *T*_max_ = 0.453	*l* = 0→30
5050 measured reflections	3 standard reflections every 120 min
4826 independent reflections	intensity decay: 2.4%

### Refinement

**Table:**

Refinement on *F*^2^	Secondary atom site location: difference Fourier map
Least-squares matrix: full	Hydrogen site location: inferred from neighbouring sites
*R*[*F*^2^ > 2σ(*F*^2^)] = 0.097	H-atom parameters constrained
*wR*(*F*^2^) = 0.317	*w* = 1/[σ^2^(*F*_o_^2^) + (0.2*P*)^2^] where *P* = (*F*_o_^2^ + 2*F*_c_^2^)/3
*S* = 1.43	(Δ/σ)_max_ = 0.019
4826 reflections	Δρ_max_ = 1.01 e Å^−^^3^
347 parameters	Δρ_min_ = −1.01 e Å^−^^3^
0 restraints	Extinction correction: *SHELXL97* (Sheldrick, 2008), Fc^*^=kFc[1+0.001xFc^2^λ^3^/sin(2θ)]^-1/4^
Primary atom site location: structure-invariant direct methods	Extinction coefficient: 0.0106 (15)

### Special details

**Table:**

Geometry. All e.s.d.'s (except the e.s.d. in the dihedral angle between two l.s. planes) are estimated using the full covariance matrix. The cell e.s.d.'s are taken into account individually in the estimation of e.s.d.'s in distances, angles and torsion angles; correlations between e.s.d.'s in cell parameters are only used when they are defined by crystal symmetry. An approximate (isotropic) treatment of cell e.s.d.'s is used for estimating e.s.d.'s involving l.s. planes.
Refinement. Refinement of *F*^2^ against ALL reflections. The weighted *R*-factor *wR* and goodness of fit *S* are based on *F*^2^, conventional *R*-factors *R* are based on *F*, with *F* set to zero for negative *F*^2^. The threshold expression of *F*^2^ > σ(*F*^2^) is used only for calculating *R*-factors(gt) *etc*. and is not relevant to the choice of reflections for refinement. *R*-factors based on *F*^2^ are statistically about twice as large as those based on *F*, and *R*-factors based on ALL data will be even larger.

### Fractional atomic coordinates and isotropic or equivalent isotropic displacement parameters (Å^2^)

**Table:**

	*x*	*y*	*z*	*U*_iso_*/*U*_eq_	
C1	0.7092 (4)	0.2838 (5)	0.52199 (18)	0.0526 (11)	
C2	0.6915 (4)	0.2131 (5)	0.4758 (2)	0.0597 (12)	
H2	0.7045	0.1269	0.4776	0.072*	
C3	0.6546 (5)	0.2695 (5)	0.42696 (19)	0.0607 (12)	
H3	0.6429	0.2220	0.3958	0.073*	
C4	0.6356 (5)	0.3958 (6)	0.4252 (2)	0.0659 (13)	
C5	0.6518 (5)	0.4666 (5)	0.4711 (2)	0.0700 (14)	
H5	0.6376	0.5525	0.4687	0.084*	
C6	0.6879 (4)	0.4148 (5)	0.5198 (2)	0.0602 (12)	
C7	1.0129 (4)	0.2164 (4)	0.58974 (17)	0.0538 (11)	
C8	1.0262 (4)	0.1581 (7)	0.54233 (19)	0.0756 (17)	
H8	0.9540	0.1354	0.5195	0.091*	
C9	1.1459 (5)	0.1329 (6)	0.5282 (2)	0.0711 (15)	
H9	1.1526	0.0907	0.4963	0.085*	
C10	1.2545 (4)	0.1677 (5)	0.5594 (2)	0.0607 (12)	
C11	1.2403 (5)	0.2234 (5)	0.6070 (3)	0.0746 (16)	
H11	1.3130	0.2441	0.6299	0.090*	
C12	1.1218 (5)	0.2505 (5)	0.6226 (2)	0.0621 (12)	
H12	1.1156	0.2912	0.6548	0.075*	
C13	0.7039 (5)	0.4992 (5)	0.5701 (2)	0.0690 (14)	
H13A	0.7856	0.4836	0.5901	0.083*	
H13B	0.6375	0.4808	0.5919	0.083*	
H13C	0.6984	0.5856	0.5594	0.083*	
C14	1.3851 (5)	0.1446 (7)	0.5412 (3)	0.0877 (18)	
H14A	1.3786	0.1505	0.5029	0.105*	
H14B	1.4145	0.0625	0.5522	0.105*	
H14C	1.4444	0.2062	0.5568	0.105*	
N1	0.8949 (4)	0.2489 (5)	0.60621 (16)	0.0686 (12)	
H1A	0.8981	0.3005	0.6325	0.082*	
O1	0.7566 (4)	0.0734 (3)	0.56948 (14)	0.0688 (10)	
O2	0.6724 (3)	0.2426 (4)	0.62092 (13)	0.0732 (11)	
Cl1	0.59229 (18)	0.46510 (19)	0.36388 (7)	0.0995 (7)	
S1	0.75361 (10)	0.20254 (13)	0.58248 (4)	0.0579 (5)	
C15	0.1219 (3)	0.6717 (4)	0.20092 (16)	0.0433 (9)	
C16	0.0837 (4)	0.6148 (4)	0.15189 (19)	0.0539 (11)	
H16	0.0822	0.5278	0.1496	0.065*	
C17	0.0488 (5)	0.6840 (5)	0.10744 (19)	0.0623 (12)	
H17	0.0223	0.6456	0.0749	0.075*	
C18	0.0536 (4)	0.8125 (5)	0.1117 (2)	0.0627 (13)	
C19	0.0895 (5)	0.8706 (4)	0.1592 (2)	0.0630 (13)	
H19	0.0899	0.9576	0.1608	0.076*	
C20	0.1252 (4)	0.8023 (4)	0.20515 (18)	0.0522 (10)	
C21	0.4121 (4)	0.6191 (4)	0.24823 (16)	0.0447 (9)	
C22	0.4261 (4)	0.5450 (4)	0.20440 (19)	0.0554 (11)	
H22	0.3661	0.4835	0.1936	0.066*	
C23	0.5307 (5)	0.5634 (4)	0.1766 (2)	0.0591 (12)	
H23	0.5398	0.5125	0.1473	0.071*	
C24	0.6215 (5)	0.6541 (4)	0.1908 (2)	0.0585 (11)	
C25	0.6048 (5)	0.7257 (5)	0.2357 (3)	0.0754 (16)	
H25	0.6653	0.7865	0.2470	0.090*	
C26	0.5028 (4)	0.7098 (5)	0.2637 (2)	0.0630 (13)	
H26	0.4943	0.7599	0.2933	0.076*	
C27	0.1653 (5)	0.8727 (4)	0.2580 (2)	0.0624 (13)	
H27A	0.2518	0.8514	0.2711	0.075*	
H27B	0.1099	0.8492	0.2839	0.075*	
H27C	0.1590	0.9614	0.2518	0.075*	
C28	0.7328 (6)	0.6763 (6)	0.1611 (3)	0.0906 (19)	
H28A	0.7071	0.7275	0.1304	0.109*	
H28B	0.7644	0.5976	0.1498	0.109*	
H28C	0.7989	0.7182	0.1839	0.109*	
N2	0.3102 (3)	0.6066 (4)	0.27950 (14)	0.0513 (9)	
H2A	0.3264	0.6176	0.3134	0.062*	
O3	0.1584 (3)	0.4483 (3)	0.23600 (13)	0.0591 (9)	
O4	0.0901 (3)	0.6035 (3)	0.29782 (12)	0.0591 (9)	
Cl2	0.01664 (19)	0.9034 (2)	0.05495 (7)	0.1068 (7)	
S2	0.16441 (9)	0.57267 (10)	0.25564 (4)	0.0463 (4)	

### Atomic displacement parameters (Å^2^)

**Table:**

	*U*^11^	*U*^22^	*U*^33^	*U*^12^	*U*^13^	*U*^23^
C1	0.039 (2)	0.067 (3)	0.051 (2)	−0.0111 (19)	0.0023 (17)	−0.014 (2)
C2	0.050 (2)	0.065 (3)	0.063 (3)	−0.008 (2)	−0.001 (2)	−0.009 (2)
C3	0.056 (3)	0.078 (3)	0.046 (2)	−0.007 (2)	−0.0031 (19)	−0.009 (2)
C4	0.051 (2)	0.087 (4)	0.059 (3)	0.000 (2)	0.006 (2)	0.007 (3)
C5	0.061 (3)	0.061 (3)	0.088 (4)	0.005 (2)	0.007 (3)	0.002 (3)
C6	0.043 (2)	0.076 (3)	0.062 (3)	−0.003 (2)	0.0063 (19)	−0.016 (2)
C7	0.048 (2)	0.065 (3)	0.046 (2)	−0.0028 (19)	−0.0032 (18)	0.0065 (19)
C8	0.041 (2)	0.137 (5)	0.045 (2)	0.005 (3)	−0.0059 (19)	−0.014 (3)
C9	0.048 (2)	0.107 (4)	0.058 (3)	0.006 (3)	0.003 (2)	−0.009 (3)
C10	0.044 (2)	0.055 (3)	0.081 (3)	0.002 (2)	0.001 (2)	0.007 (2)
C11	0.051 (3)	0.068 (3)	0.099 (4)	−0.006 (2)	−0.017 (3)	−0.010 (3)
C12	0.059 (3)	0.058 (3)	0.065 (3)	−0.008 (2)	−0.009 (2)	−0.005 (2)
C13	0.060 (3)	0.069 (3)	0.080 (3)	0.001 (2)	0.017 (2)	−0.030 (3)
C14	0.047 (3)	0.092 (4)	0.124 (5)	0.004 (3)	0.010 (3)	−0.003 (4)
N1	0.047 (2)	0.110 (3)	0.048 (2)	−0.008 (2)	0.0038 (16)	−0.024 (2)
O1	0.076 (2)	0.073 (2)	0.058 (2)	−0.0125 (18)	0.0113 (17)	0.0022 (17)
O2	0.0535 (18)	0.124 (3)	0.0464 (18)	−0.0222 (19)	0.0228 (14)	−0.0159 (18)
Cl1	0.0931 (11)	0.1265 (15)	0.0774 (10)	0.0192 (10)	0.0034 (8)	0.0262 (9)
S1	0.0474 (7)	0.0810 (9)	0.0457 (7)	−0.0131 (5)	0.0077 (5)	−0.0067 (5)
C15	0.0366 (18)	0.048 (2)	0.046 (2)	0.0015 (16)	0.0062 (15)	−0.0090 (17)
C16	0.051 (2)	0.053 (2)	0.058 (3)	−0.0032 (19)	0.0037 (19)	−0.009 (2)
C17	0.062 (3)	0.076 (3)	0.048 (2)	0.009 (2)	0.004 (2)	−0.003 (2)
C18	0.050 (2)	0.083 (3)	0.056 (3)	0.020 (2)	0.010 (2)	0.008 (2)
C19	0.055 (3)	0.051 (3)	0.085 (4)	0.016 (2)	0.016 (2)	0.003 (2)
C20	0.049 (2)	0.048 (2)	0.060 (3)	0.0078 (18)	0.0066 (19)	−0.0085 (19)
C21	0.0405 (19)	0.048 (2)	0.045 (2)	0.0003 (17)	0.0023 (16)	0.0022 (17)
C22	0.056 (3)	0.048 (2)	0.063 (3)	−0.0056 (19)	0.013 (2)	−0.007 (2)
C23	0.066 (3)	0.051 (2)	0.064 (3)	0.004 (2)	0.020 (2)	−0.004 (2)
C24	0.056 (2)	0.053 (2)	0.070 (3)	0.002 (2)	0.023 (2)	0.004 (2)
C25	0.057 (3)	0.070 (3)	0.103 (4)	−0.019 (2)	0.026 (3)	−0.027 (3)
C26	0.052 (2)	0.071 (3)	0.067 (3)	−0.009 (2)	0.013 (2)	−0.023 (3)
C27	0.073 (3)	0.042 (2)	0.070 (3)	0.010 (2)	−0.001 (2)	−0.019 (2)
C28	0.086 (4)	0.082 (4)	0.114 (5)	−0.015 (3)	0.053 (4)	−0.006 (4)
N2	0.0413 (17)	0.070 (2)	0.0421 (18)	−0.0051 (16)	0.0027 (14)	0.0000 (16)
O3	0.065 (2)	0.0463 (17)	0.066 (2)	−0.0074 (14)	0.0089 (15)	0.0000 (14)
O4	0.0509 (17)	0.082 (2)	0.0487 (17)	−0.0144 (15)	0.0230 (13)	−0.0088 (15)
Cl2	0.1074 (13)	0.1270 (15)	0.0883 (12)	0.0445 (11)	0.0214 (10)	0.0449 (10)
S2	0.0427 (6)	0.0495 (6)	0.0469 (7)	−0.0047 (4)	0.0062 (4)	−0.0025 (4)

### Geometric parameters (Å, °)

**Table:**

C1—C2	1.381 (7)	C15—C16	1.392 (6)
C1—C6	1.416 (7)	C15—C20	1.399 (6)
C1—S1	1.768 (5)	C15—S2	1.753 (4)
C2—C3	1.384 (7)	C16—C17	1.356 (7)
C2—H2	0.9300	C16—H16	0.9300
C3—C4	1.362 (8)	C17—C18	1.377 (8)
C3—H3	0.9300	C17—H17	0.9300
C4—C5	1.374 (8)	C18—C19	1.360 (7)
C4—Cl1	1.726 (5)	C18—Cl2	1.734 (5)
C5—C6	1.360 (8)	C19—C20	1.381 (7)
C5—H5	0.9300	C19—H19	0.9300
C6—C13	1.547 (6)	C20—C27	1.543 (6)
C7—C8	1.369 (7)	C21—C22	1.381 (6)
C7—C12	1.382 (6)	C21—C26	1.384 (6)
C7—N1	1.401 (6)	C21—N2	1.412 (5)
C8—C9	1.378 (7)	C22—C23	1.389 (7)
C8—H8	0.9300	C22—H22	0.9300
C9—C10	1.362 (7)	C23—C24	1.379 (7)
C9—H9	0.9300	C23—H23	0.9300
C10—C11	1.363 (8)	C24—C25	1.394 (7)
C10—C14	1.522 (7)	C24—C28	1.484 (7)
C11—C12	1.384 (8)	C25—C26	1.365 (7)
C11—H11	0.9300	C25—H25	0.9300
C12—H12	0.9300	C26—H26	0.9300
C13—H13A	0.9600	C27—H27A	0.9600
C13—H13B	0.9600	C27—H27B	0.9600
C13—H13C	0.9600	C27—H27C	0.9600
C14—H14A	0.9600	C28—H28A	0.9600
C14—H14B	0.9600	C28—H28B	0.9600
C14—H14C	0.9600	C28—H28C	0.9600
N1—S1	1.615 (4)	N2—S2	1.623 (3)
N1—H1A	0.8600	N2—H2A	0.8600
O1—S1	1.418 (4)	O3—S2	1.415 (3)
O2—S1	1.433 (3)	O4—S2	1.432 (3)
			
C2—C1—C6	120.2 (5)	C16—C15—C20	120.3 (4)
C2—C1—S1	116.9 (4)	C16—C15—S2	117.1 (3)
C6—C1—S1	122.9 (3)	C20—C15—S2	122.7 (3)
C1—C2—C3	120.5 (5)	C17—C16—C15	121.1 (4)
C1—C2—H2	119.8	C17—C16—H16	119.4
C3—C2—H2	119.8	C15—C16—H16	119.4
C4—C3—C2	118.9 (5)	C16—C17—C18	118.3 (5)
C4—C3—H3	120.5	C16—C17—H17	120.8
C2—C3—H3	120.5	C18—C17—H17	120.8
C3—C4—C5	120.9 (5)	C19—C18—C17	121.8 (5)
C3—C4—Cl1	118.4 (4)	C19—C18—Cl2	118.9 (4)
C5—C4—Cl1	120.7 (5)	C17—C18—Cl2	119.3 (4)
C6—C5—C4	121.9 (5)	C18—C19—C20	121.1 (5)
C6—C5—H5	119.0	C18—C19—H19	119.5
C4—C5—H5	119.0	C20—C19—H19	119.5
C5—C6—C1	117.6 (5)	C19—C20—C15	117.4 (4)
C5—C6—C13	119.6 (5)	C19—C20—C27	119.0 (4)
C1—C6—C13	122.8 (5)	C15—C20—C27	123.6 (4)
C8—C7—C12	118.6 (5)	C22—C21—C26	119.4 (4)
C8—C7—N1	123.9 (4)	C22—C21—N2	123.6 (4)
C12—C7—N1	117.5 (4)	C26—C21—N2	117.1 (4)
C7—C8—C9	120.5 (4)	C21—C22—C23	119.3 (4)
C7—C8—H8	119.8	C21—C22—H22	120.3
C9—C8—H8	119.8	C23—C22—H22	120.3
C10—C9—C8	122.0 (5)	C24—C23—C22	122.4 (4)
C10—C9—H9	119.0	C24—C23—H23	118.8
C8—C9—H9	119.0	C22—C23—H23	118.8
C9—C10—C11	117.0 (5)	C23—C24—C25	116.4 (4)
C9—C10—C14	120.7 (5)	C23—C24—C28	123.2 (5)
C11—C10—C14	122.3 (5)	C25—C24—C28	120.4 (5)
C10—C11—C12	122.6 (5)	C26—C25—C24	122.3 (5)
C10—C11—H11	118.7	C26—C25—H25	118.8
C12—C11—H11	118.7	C24—C25—H25	118.8
C11—C12—C7	119.2 (5)	C25—C26—C21	120.1 (5)
C11—C12—H12	120.4	C25—C26—H26	119.9
C7—C12—H12	120.4	C21—C26—H26	119.9
C6—C13—H13A	109.5	C20—C27—H27A	109.5
C6—C13—H13B	109.5	C20—C27—H27B	109.5
H13A—C13—H13B	109.5	H27A—C27—H27B	109.5
C6—C13—H13C	109.5	C20—C27—H27C	109.5
H13A—C13—H13C	109.5	H27A—C27—H27C	109.5
H13B—C13—H13C	109.5	H27B—C27—H27C	109.5
C10—C14—H14A	109.5	C24—C28—H28A	109.5
C10—C14—H14B	109.5	C24—C28—H28B	109.5
H14A—C14—H14B	109.5	H28A—C28—H28B	109.5
C10—C14—H14C	109.5	C24—C28—H28C	109.5
H14A—C14—H14C	109.5	H28A—C28—H28C	109.5
H14B—C14—H14C	109.5	H28B—C28—H28C	109.5
C7—N1—S1	128.8 (3)	C21—N2—S2	124.3 (3)
C7—N1—H1A	115.6	C21—N2—H2A	117.8
S1—N1—H1A	115.6	S2—N2—H2A	117.8
O1—S1—O2	118.6 (2)	O3—S2—O4	118.0 (2)
O1—S1—N1	109.8 (2)	O3—S2—N2	109.8 (2)
O2—S1—N1	104.9 (2)	O4—S2—N2	104.56 (19)
O1—S1—C1	106.8 (2)	O3—S2—C15	107.08 (19)
O2—S1—C1	108.5 (2)	O4—S2—C15	109.5 (2)
N1—S1—C1	107.9 (2)	N2—S2—C15	107.42 (19)
			
C6—C1—C2—C3	0.9 (7)	C20—C15—C16—C17	−0.2 (7)
S1—C1—C2—C3	178.9 (4)	S2—C15—C16—C17	179.8 (4)
C1—C2—C3—C4	−0.2 (7)	C15—C16—C17—C18	0.9 (7)
C2—C3—C4—C5	−0.5 (8)	C16—C17—C18—C19	−1.4 (8)
C2—C3—C4—Cl1	178.9 (4)	C16—C17—C18—Cl2	177.0 (4)
C3—C4—C5—C6	0.5 (8)	C17—C18—C19—C20	1.3 (8)
Cl1—C4—C5—C6	−178.9 (4)	Cl2—C18—C19—C20	−177.2 (4)
C4—C5—C6—C1	0.2 (8)	C18—C19—C20—C15	−0.5 (7)
C4—C5—C6—C13	−179.7 (5)	C18—C19—C20—C27	179.9 (4)
C2—C1—C6—C5	−0.9 (7)	C16—C15—C20—C19	0.0 (6)
S1—C1—C6—C5	−178.8 (4)	S2—C15—C20—C19	180.0 (3)
C2—C1—C6—C13	179.0 (4)	C16—C15—C20—C27	179.6 (4)
S1—C1—C6—C13	1.1 (6)	S2—C15—C20—C27	−0.5 (6)
C12—C7—C8—C9	0.8 (9)	C26—C21—C22—C23	−0.3 (7)
N1—C7—C8—C9	177.9 (6)	N2—C21—C22—C23	−179.1 (4)
C7—C8—C9—C10	−2.1 (10)	C21—C22—C23—C24	−0.6 (7)
C8—C9—C10—C11	3.3 (9)	C22—C23—C24—C25	1.4 (8)
C8—C9—C10—C14	−176.7 (6)	C22—C23—C24—C28	−179.1 (5)
C9—C10—C11—C12	−3.4 (8)	C23—C24—C25—C26	−1.5 (9)
C14—C10—C11—C12	176.6 (5)	C28—C24—C25—C26	179.0 (6)
C10—C11—C12—C7	2.2 (9)	C24—C25—C26—C21	0.7 (9)
C8—C7—C12—C11	−0.8 (8)	C22—C21—C26—C25	0.3 (8)
N1—C7—C12—C11	−178.1 (5)	N2—C21—C26—C25	179.1 (5)
C8—C7—N1—S1	14.5 (8)	C22—C21—N2—S2	−37.0 (6)
C12—C7—N1—S1	−168.4 (4)	C26—C21—N2—S2	144.2 (4)
C7—N1—S1—O1	39.5 (5)	C21—N2—S2—O3	67.9 (4)
C7—N1—S1—O2	167.9 (5)	C21—N2—S2—O4	−164.5 (4)
C7—N1—S1—C1	−76.5 (5)	C21—N2—S2—C15	−48.3 (4)
C2—C1—S1—O1	−2.3 (4)	C16—C15—S2—O3	4.1 (4)
C6—C1—S1—O1	175.6 (4)	C20—C15—S2—O3	−175.9 (3)
C2—C1—S1—O2	−131.2 (4)	C16—C15—S2—O4	−125.0 (3)
C6—C1—S1—O2	46.7 (4)	C20—C15—S2—O4	55.0 (4)
C2—C1—S1—N1	115.6 (4)	C16—C15—S2—N2	122.0 (3)
C6—C1—S1—N1	−66.5 (4)	C20—C15—S2—N2	−58.0 (4)

### Hydrogen-bond geometry (Å, °)

**Table:**

*D*—H···*A*	*D*—H	H···*A*	*D*···*A*	*D*—H···*A*
N1—H1A···O4^i^	0.86	2.02	2.874 (5)	171.
N2—H2A···O2^i^	0.86	2.23	2.968 (5)	144.

Symmetry codes: (i) −*x*+1, −*y*+1, −*z*+1.

## References

[bb1] Enraf–Nonius (1996). *CAD-4-PC* Enraf–Nonius, Delft, The Netherlands.

[bb2] Gelbrich, T., Hursthouse, M. B. & Threlfall, T. L. (2007). *Acta Cryst.* B**63**, 621–632. 10.1107/S010876810701395X 17641433

[bb3] Gowda, B. T., Foro, S., Nirmala, P. G., Babitha, K. S. & Fuess, H. (2009). *Acta Cryst.* E**65**, o476. 10.1107/S1600536809003845 PMC2968641 21582145

[bb4] Gowda, B. T., Foro, S., Nirmala, P. G. & Fuess, H. (2010). *Acta Cryst.* E**66**, o2000. 10.1107/S1600536810026930 PMC3007535 21588314

[bb5] North, A. C. T., Phillips, D. C. & Mathews, F. S. (1968). *Acta Cryst.* A**24**, 351–359.

[bb6] Perlovich, G. L., Tkachev, V. V., Schaper, K.-J. & Raevsky, O. A. (2006). *Acta Cryst.* E**62**, o780–o782.

[bb7] Savitha, M. B. & Gowda, B. T. (2006). *Z. Naturforsch. Teil A*, **60**, 600–606.

[bb8] Sheldrick, G. M. (2008). *Acta Cryst.* A**64**, 112–122. 10.1107/S0108767307043930 18156677

[bb9] Spek, A. L. (2009). *Acta Cryst.* D**65**, 148–155. 10.1107/S090744490804362X PMC2631630 19171970

[bb10] Stoe & Cie (1987). *REDU4* Stoe & Cie GmbH, Darmstadt, Germany.

